# Protective Role of Glutathione and Nitric Oxide Production in the Pathogenesis of Pterygium

**DOI:** 10.1155/2020/9638763

**Published:** 2020-08-29

**Authors:** Fidelina Parra, Alexander Kormanovski, Gustavo Guevara-Balcazar, María del Carmen Castillo-Hernández, Antonio Franco-Vadillo, Mireille Toledo-Blas, Rosa Adriana Jarillo-Luna, Eleazar Lara-Padilla

**Affiliations:** Departamento de Posgrado e Investigación, Escuela Superior de Medicina, Instituto Politécnico Nacional, Mexico City, Mexico

## Abstract

**Objective:**

In the pathogenesis of pterygium, the protective role of glutathione and nitric oxide production is unclear. These are important factors for homeostasis in the redox state of cells. The aim of this study was to determine the levels of these and related parameters in pterygium tissue. *Patients and Methods*. The study sample consisted of 120 patients diagnosed with primary or recurrent pterygium. Five groups of tissue samples were examined: control, primary pterygium, recurrent pterygium, and two groups of primary pterygium given a one-month NAC presurgery treatment (topical or systemic). The levels of endothelial nitric oxide synthase (eNOS), nitric oxide (NO), 3-nitrotyrosine (3NT), reduced and oxidized glutathione (GSH and GSSG), and catalase (CAT) were evaluated in tissue homogenates.

**Results:**

Compared with the control, decreased levels of eNOS, NO, and 3-nitrotyrosine as well as the degree of oxidation of GSH (GSSG%) were observed in primary and recurrent pterygium. 3-Nitrotyrosine and GSSG% were reduced in the other pterygium groups. GSH and CAT were enhanced in recurrent pterygium and systemic-treated primary pterygium but were unchanged for topical-treated primary pterygium. There was a strong positive correlation of eNOS with NO and 3NT, GSSG% with NO and 3NT, and GSH with GSSG and CAT. Women showed a higher level of GSH and catalase in primary pterygium, whereas a lower level of GSH and a higher level of NO in recurrent pterygium.

**Conclusion:**

The results are congruent with the following proposed sequence of events leading to a protective response of the organism during the pathogenesis of primary pterygium: a decreased level of eNOS provokes a decline in the level of NO in pterygium tissue, which then leads to reduced S-nitrosylation of GSH or other thiols and possibly to the modulation of the intracellular level of GSH through synthesis and/or mobilization from other tissues.

## 1. Introduction

Pterygium is triggered by a degenerative lesion in the conjunctiva and cornea of the eye and proceeds to fibrous tissue growth on the surface of the cornea. Surgery is the principal treatment, leading in some cases to recurrence. The mechanisms of pathogenesis have been discussed in several reviews [1–3]. The key factor in the etiology of pterygium is considered to be chronic exposure to UV radiation [4, 5], resulting in oxidative stress. In the long run, this condition can cause damage to DNA, other critical biological molecules, and the structure of the cell membrane. Indeed, this type of cell damage can contribute to the pathogenesis of different ocular diseases. The mechanisms of the protective response of the organism during the development of pterygium are not clear.

Various molecules contribute to oxidative stress, including nitric oxide (NO) at an elevated concentration and derivatives of the same (e.g., peroxynitrite). Peroxynitrite is very potent oxidant that reacts with tyrosine residues of proteins to form 3-nitrotyrosine (3NT). 3NT serves as an indicator of nitrosative stress. However, these molecules are also important for cell signaling, involved in mechanisms for modulating and adapting to oxidative stress in a multitude of tissues. The synthesis and/or redistribution of glutathione (GSH) is one such possible mechanism. Reports on NO in pterygium tissue are contradictory, as both low [6] and high levels [7] have been found, the latter reflecting probably the quantity of women in the study (by the higher levels of NO in women, compared with men). The overall effect of NO and related molecules revolves around the balance between the positive physiological ramifications of their signaling and the negative consequences of oxidative stress.

There are no published data, to our knowledge, on the level of GSH in pterygium tissue. This antioxidant is synthesized in all tissues but in notably larger quantities in the liver. Although three constitutive amino acids (cysteine, glutamate, and glycine) participate in the synthesis of GSH, the limiting factor is cysteine. Under healthy conditions, the concentration of GSH reaches millimolar levels in the cornea and micromolar levels in other ocular tissues (as well as in fluids) [8, 9]. Micromolar concentrations of reduced GSH and oxidized GSH (GSSG) have been described in retinoblastoma [10]. NO reportedly affects the level of GSH in the liver of mice with induced endotoxemia: a reduction in NO stimulates GSH synthesis and on the contrary [11]. The relation NO-GSH was observed in primary cultured conjunctival epithelial cell layers from rabbits [12] and in retinal pigment epithelial cells [13]. GSH has a strong affinity for NO, and S-nitrosoglutathione is the primary intermediary agent in the production of other nitrosothiols that regulate metabolism [14, 15]. This suggests that the most relevant effect of NO in the pathogenesis of pterygium may be on the synthesis and/or redistribution of GSH in ocular tissues.

N-Acetylcysteine (NAC) is derived from the amino acid L-cysteine and is used as a precursor of GSH synthesis in respiratory tract diseases [16]. By modulating oxidative stress, L-cysteine indirectly exerts an antiviral and anti-inflammatory effect, inhibits angiogenesis, and inhibits cancer cell growth in different diseases, including ocular tissues [17, 18]. The aim of the present study was to evaluate the levels of GSH, GSSG, NO, 3-nitrotyrosine (3NT), catalase (CAT), and endothelial nitric oxide synthase (eNOS) in tissues samples of pterygium representing four conditions: untreated primary pterygium, recurrent pterygium, and primary pterygium treated with NAC, either systemic or topic.

## 2. Patients and Methods

### 2.1. Study Design

This study was conducted in the Hospital “Nuestra Señora de la Luz” specialized in ophthalmology and the High Medical School of National Polytechnic Institute (Mexico). This study was performed in accordance with the Helsinki Declaration. Ethical approval was obtained from the Hospital Ethics Committee (No. 3-2015 FHNSL-CE). Informed consent was obtained from each of the participants.

### 2.2. Study Population

A total of 120 patients (54 men and 66 women) took part in the study, selected from individuals who went to the “Nuestra Señora de la Luz” Hospital for pterygium resection surgery from 2015 to 2019 and had been given a clinical diagnosis of primary or recurrent pterygium. The tissue samples evaluated represent five conditions: the control (healthy conjunctiva) and four patient groups, including untreated primary pterygium, recurrent pterygium, and primary pterygium treated topically or systemically with NAC. The following were exclusion criteria: (1) history of disease or trauma to the ocular surface involving the sampling area; (2) ocular surgery during the three months prior to the pending pterygium surgery; (3) oral or topical immunosuppressive treatment during four weeks before the pending pterygium surgery; (4) systemic diseases such as diabetes, rheumatoid arthritis, another autoimmune disease, or neoplasia; and (5) chronic addictions to substances (e.g., tobacco or alcohol). Finally, during the four years of the study, 28 patients with primary pterygium (12 women and 16 men) were excluded.

### 2.3. Tissue Samples

The pterygium samples were obtained during pterygium resection surgery. The surgery was performed by dissecting the head of the pterygium of the cornea, followed by resection of the body in a block. During the process, the conjunctiva was taken together with the subconjunctival fibrovascular tissue. The bare sclera was covered with a conjunctival autograft taken from the upper or lower conjunctiva and fixed with nylon 10.0 sutures in separate stitches. The control samples were obtained from the resection of conjunctiva resulting from extracapsular cataract extraction. During this resection, conjunctiva contained minimal quantity (10–20 mg) of healthy tissue that was separated for control samples. The tissue extracted was stored in 2 mL flat-bottom Eppendorf tubes and immediately frozen in liquid nitrogen and then stored at −70°C to await processing.

### 2.4. Treatments

For systemic treatment of primary pterygium, NAC was administered at 600 mg/day for one month prior to surgery. For respiratory diseases, the same dose is commonly used in systemic treatments aimed at increasing the level of GSH in tissues [16]. Topical treatment of primary pterygium, also lasting one month before surgery, involved the application of one drop of a 10% solution of NAC four times per day. The treatment groups do not represent a clinical trial. Rather, they are a scientific instrument for exploring the origin of high levels of GSH in recurrent pterygium. The reason for choosing NAC as the present treatment is of its prior use as a therapy for different pathologies including ocular disorders.

### 2.5. Samples Processing

Samples were processed periodically, when approximately 20 had accumulated, resulting in an average storage time of 2–3 weeks after collection. Samples of homogenates were obtained by placing the tissue in a 30 mmol cold phosphate buffer solution (pH 7.2) and adding 0.1% of Triton 100 (1 mg of tissue per 10 *μ*L buffer). Tissues were homogenized and centrifuged at 10,000 rpm for 15 min at 4°C, and the supernatants were stored at −70°C to be processed within two weeks. Regarding the tissue homogenates, assay kits (Cayman Chemical, MI, USA) were employed for measurement of total proteins (TP, No. 704002), NO (nitrate/nitrite colorimetric assay kit, No. 780001), total reduced and oxidized GSH (glutathione assay kit, No. 703002), and CAT (CAT assay kit, No. 707002). The level of 3NT (3-nitrotyrosine ELISA kit, No. ab116691; Abcam, UK) was established in homogenates by the enzyme-linked immunosorbent assay (ELISA). The values of NO, GSH, GSSG, and 3NT are expressed as nmol/mg of TP/mL, corresponding to a *μ*mol concentration. The degree of GSH oxidation was calculated by the following equation: GSSG% = GSSG/2GSH × 100.

### 2.6. Western Blot Assay

From the different groups with pterygium and control group, 100 *μ*g of protein was subjected to 10% SDS-PAGE under nonreducing conditions and transferred to polyvinylidene fluoride membranes (Immobilon PVDF, 0.45 *μ*m; Millipore, USA). The membranes were then blocked with 5% albumin serum bovine in TBS and 0.1% Tween 20 (TBS-T, pH 7.4) for two hours. Subsequently, they were washed three times with TBS-T and incubated with the primary antibody for eNOS (Cat. SC-5302) at 4°C for 18 h under continuous agitation. After adequately washing with TBS-T, the membranes were incubated with the secondary antibody (Cat. SC-516102) for 2 h under constant agitation. Detection was then carried out by the enhanced chemiluminescence method (Western Blotting Luminol Reagent, Cat. 2048; Santa Cruz, CA, USA). Membranes were photographed, and the image was digitalized to perform densitometric analysis using the Image Studio Lite software (LI-COR Biosciences). The relative presence of each protein was normalized with *β*-actin as the housekeeping protein. Before implementation of Western blot, the study groups were separated into subgroups of six samples each. Three groups had an average of 3 × 6 samples each: the control, recurrent pterygium, and primary pterygium with systemic treatment. The untreated primary pterygium group had 6 × 6 determinations, and the topic-treated primary pterygium group, 4 × 6 determinations.

### 2.7. Statistical Analysis

Data were expressed as the mean values ± standard deviations for each group and examined using the GraphPad Prism software, version 6 (GraphPad Software Inc., La Jolla, CA, USA). The results were analyzed by ANOVA, followed by Tukey post hoc test, considering significance at *p* < 0.05. The bivariate Pearson correlation was also used to compare the parameters between groups.

## 3. Results

### 3.1. Population Data

The data of the participants are presented in Table 1.

The average age of individuals in the recurrent pterygium group was lower than that in the control group. Upon comparing the primary pterygium group with the other pathological groups, some moderate differences were detected (principally in men). Taking into account all patients with primary pterygium who used the service of the hospital (including those excluded from the study), the percentage of recurrence was 17% for all patients, 19% for men, and 16% for women. Among the pterygium groups with a limited quantity of patients, recurrence occurred in 2 with systemic treatment (1 man and 1 woman, 13% and 11%, respectively, of the treatment group considered by gender) and in 3 with topical treatment (1 man and 2 women, 9% and 11%, respectively, of the treatment group by gender). However, it is incorrect to make a statistical comparison of these data with those from all patients because these recurrence groups were only from the last year of the study.

### 3.2. Oxidant/Antioxidant Response in All Patients

**Figure 1:**
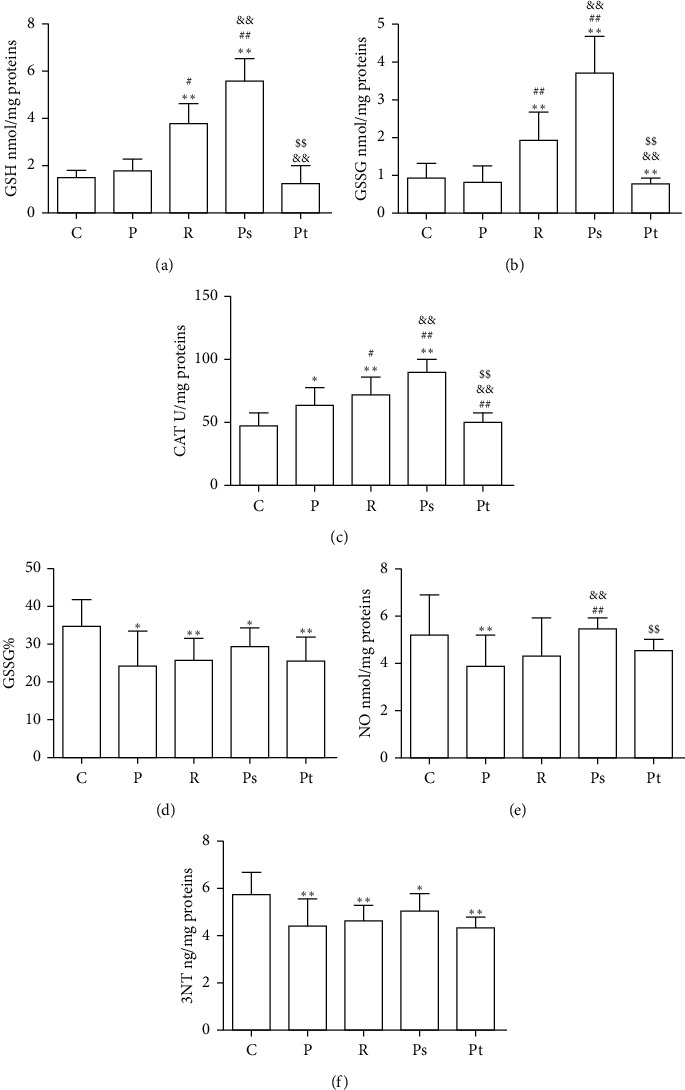
Oxidant/antioxidant response in all patients regardless of gender (mean ± SD). Levels of reduced (a) and oxidized GSH (b), CAT (c), degree of GSH oxidation (d), NO (e), and 3-nitrotyrosine (f) in the control group (C), primary pterygium (P), recurrent pterygium (R), and primary pterygium with systemic (Ps) or topical (Pt) pretreatment in all patients regardless of gender. ^*∗*^*p* < 0.05 and ^*∗∗*^*p* < 0.01 compared with group C. ^#^*p* < 0.05 and ^##^*p* < 0.01 compared with the primary pterygium group. ^&^*p* < 0.05 and ^&&^*p* < 0.01 compared with group R. ^$$^*p* < 0.01 compared groups with treatments.

Data on the level of reduced GSH, GSSG, GSSG%, CAT, NO, and 3NT (Figure 1) for all patients (regardless of gender) were obtained.

Surprisingly, compared with the control, there was a significantly higher concentration of GSH in recurrent pterygium tissue, and an even greater increase in systemic-treated primary pterygium tissue. On contrary, the level of GSH was not significantly different when comparing topical-treated primary pterygium with either the control or untreated primary pterygium. A similar pattern was observed for the concentration of GSSG and CAT. The value of GSSG%, on the other hand, was low in all groups compared with the control.

GSSG% was herein found to be relatively high in the ocular tissue of all groups. The value for the control group was about 35%, and for the pathological groups, around 25%, in contrast to the 1-2% reported in other tissues of the body. In relation to the control group, the level of 3NT was significantly lower in all pathological groups, whereas only in the untreated primary pterygium did NO decrease.

### 3.3. eNOS Response in All Patients

**Figure 2:**
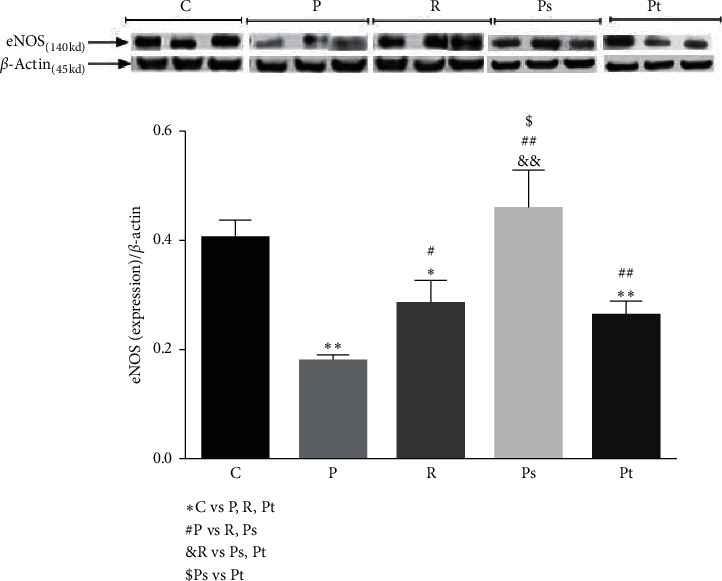
Response of eNOS in all patients regardless of gender. Levels of eNOS activity in the control group (C), primary pterygium (P), recurrent pterygium (R), and primary pterygium with systemic (Ps) or topical (Pt) pretreatment in all patients regardless of gender. ^*∗*^*p* < 0.05 and ^*∗∗*^*p* < 0.01 compared with group C. ^#^*p* < 0.05 and ^##^*p* < 0.01 compared with primary pterygium group. ^&^*p* < 0.05 and ^&&^*p* < 0.01 compared with group R. ^$^*p* < 0.05 compared groups with treatments.

Figure 2 presents the activity of eNOS by groups in all patients regardless of gender.

There was a decrease in eNOS activity in untreated primary pterygium and a smaller reduction in topical-treated primary pterygium as well as recurrent pterygium. There was a decrease in eNOS activity in untreated primary pterygium and a smaller reduction in topical-treated primary pterygium as well as recurrent pterygium. No change in eNOS was detected with systemic-treated primary pterygium.

Compared with men, women exhibited a greater concentration of GSH in untreated primary pterygium and a lower concentration in systemic-treated primary pterygium. For GSSG and GSSG%, no gender differences existed. The level of CAT was higher in women than men in the recurrent pterygium group, which coincides with an increased presence of NO in these women (data not presented).

### 3.4. Bivariate Pearson Correlation

The bivariate Pearson correlation, considering all participants together as well as men and women separately (Table 2), analyzed the behavioral pattern of parameters between groups.

Considering all patients, the correlations are positive for GSH with CAT and GSSG, GSSG% with NO and 3NT, and eNOS with NO and 3NT. Examining men and women separately, the same positive association was found for GSH with CAT and GSSG. On the other hand, GSSG% only correlated with NO in men and 3NT in women.

## 4. Discussion

The importance of GSH for the protection of eye tissue has been well documented [8]. For the maintenance of redox homeostasis in the eye, GSH plays an essential role in cells and tissues as well as the tear film. GSH is thought to participate in intracellular repair of protein damaged by oxidative stress [13]. GSH moves from the conjunctiva towards the tear film by means of the Na^+^-independent efflux system [19–21], which is considered an essential mechanism of H_2_O_2_ neutralization in the latter fluid [22]. As is true for diverse tissues, all eye tissues are supplied with GSH through the bloodstream. The liver contributes to redox homeostasis in a multitude of tissues by releasing GSH into the bloodstream. This organ is capable of producing a greater amount of GSH than any other tissue in the body [23], leading to a high concentration in its efflux.

Since GSH cannot pass directly into cells, it must be metabolized into its constituent amino acids, which can pass through the membrane and serve as substrates for intracellular GSH resynthesis. Therefore, it is essential for its constituents to be transported through cell membranes so that redox homeostasis can be maintained. The limiting factor for GSH synthesis is normally the availability of cysteine [24]. Hence, the use of NAC as a cysteine precursor is justified for the management of some pathologies, including ocular disorders. The transport of L-cysteine or L-glutamate across cell membranes takes place in several types of eye cells (e.g., human retinal pigment epithelium), increasing the uptake of L-cystine (the oxidized form of cysteine) and thus leading to a higher level of intracellular GSH. Moreover, nitrosative stress enhances the uptake of L-cysteine and the enzyme activity of *ɣ*-glutamylcysteine synthase (GCS), which are responsible for the synthesis of GSH in rabbit conjunctival epithelial cell layers [12], where the behavior pattern of the level of GSH is contrary to that found in the liver [11].

In the present study, there was a low level of eNOS, NO, and 3NT (indicator of nitrosative stress) in primary and recurrent pterygium tissue versus the control group. In our previous research, no significant change was detected in lipoperoxidation (TBARS) when comparing both pathological groups to healthy tissues [6]. Hence, the decrease of nitrosative stress probably diminishes the rate of inactivation of GSH and CAT in recurrent pterygium, thus promoting a greater level of these parameters. In the same sense, the positive correlation of CAT with GSH in the pathological groups suggests that an enhanced level of the latter in these tissues would trigger a reduction in the rate of inactivation of the former. The strong positive correlation found in the different groups between eNOS activity and the levels of NO and 3NT confirms the regulation of NO production in pterygium tissue by eNOS. In the untreated primary pterygium tissue, the concentration of NO and 3NT decreased without significantly affecting a total level of total GSH (GSH + GSSG). Surprisingly, an elevated concentration of GSH was observed in recurrent pterygium and an even greater increase in systemic-treated primary pterygium, relative to the control group or untreated primary pterygium. It is possible that in patients with recurrent pterygium, the conjunctiva needs higher levels of GSH to maintain homeostasis in the redox state of its cells and that by some mechanism it is synthesized and/or mobilized from other tissues. When comparing women with men, the former had elevated GSH and CAT in the untreated primary pterygium group, as well as a lower level of GSH and a higher level of NO in the recurrent pterygium group. There was a positive correlation in the groups between GSSG% and 3NT for women, and between GSSG% and NO for men.

In a previous study [6], we observed a significant decline in the total antioxidant status (TAS) in recurrent pterygium compared with the control and primary pterygium. Since the decrease in antioxidant enzyme activity was not significant (except CAT), we made the supposition that this change in TAS was determined by the nonenzymatic part of the total antioxidant capacity.

As aforementioned, a reduction in NO stimulates GSH synthesis in tissues. During endotoxemia in animal models, the decrease of NO in liver increased the level of GSH and vice versa [11]. Regarding the present study, diminished levels of NO and 3NT could have stimulated GSH synthesis in primary pterygium tissue, but this was not observed.

The drop in the percentage of GSH oxidation (GSSG%) from 35% to 25% in this group may be related to a mechanism of equilibrium consisting of a diminished rate of GSH oxidation and an increased supply of reduced GSH. The former could be explained by a decrease in nitrosative stress and the latter by the stimulation of synthesis of GSH and/or its redistribution from nearby tissue (the cornea). The efflux of GSH from the conjunctiva to tear film has been described [21, 22]. No reports could be found in relation to an efflux of GSH from the cornea to extracellular space and after in conjunctiva, though such efflux would not be surprising, given the millimolar concentration in the cornea vs micromolar in conjunctiva.

The benefits of topical therapy involving GSH or its prodrugs have mainly been reported in relation to tissues showing a low level of this antioxidant, such as diseased eye tissue [8, 12, 25]. A positive outcome has been documented as a result of the administration of NAC to patients with diabetic retinopathy [26]. Additionally, NAC provided protection against alloxan-induced diabetes in mice by boosting the synthesis of GSH in platelets [27] and improved axis-related symptoms in patients with dry eye syndrome [18]. In the current effort, topical treatment of pterygium did not modify the level of GSH compared with the control group or untreated primary pterygium. The effect of topical treatment indicates that cysteine does not directly enter pterygium tissue. One or more of three mechanisms likely cause the enhanced level of GSH in recurrent pterygium tissue: a decrease in nitrosative stress, the stimulation of GSH synthesis, and/or the efflux (mobilization) of GSH from nearby tissue with a higher concentration (i.e., the cornea). The nearby tissue can recover the loss of GSH from the bloodstream, which in turn is supplied by the liver. Thus, systemic presurgery treatment with NAC may stimulate the synthesis of GSH in the liver, which would result in its redistribution to diverse tissues. Unlike systemic treatment, topical treatment showed no effect on intracellular levels of GSH in pterygium tissue, perhaps, because the direct transport of cysteine into pathological tissue is impeded.

In the present study, a systemic (but not topical) presurgery NAC-based treatment significantly increased the level of GSH in patients with pterygium. This should certainly be interpreted as a positive effect.

NO is a free radical with a short life but a great capacity to penetrate cells. When NO reacts with proteins (S-nitrosylation), it is thought to acquire a longer lifetime. S-Nitrosylation is herein determined as the NO-induced modification of protein thiols [14]. GSH has a strong affinity for NO, and S-nitrosoglutathione is the main intermediate in the formation of other nitrosothiols that regulate metabolism [14, 15]. In this case, nitrosothiols are NO stabilizers and potential carrier molecules, rather than a product of NO activity [15]. A possible reaction that releases NO from S-nitrosoglutathione is as follows: GSNO = GSSG + 2NO [28]. However, GSNO is S-nitrosoglutathione. As can be appreciated, the release of NO is accompanied by GSSG production. Indeed, NO presently showed a correlation with the degree of oxidation of GSH (GSSG%). The distinct nitrosothiols have a different half-life in water. The half-life of S-nitrosoglutathione is expressed in hours and that of S-nitrosocysteine and S-nitroso-N-acetylcysteine in minutes. These compounds are able to affect the synthesis of intracellular GSH as well as its efflux from tissues with a relatively high concentration (i.e., the liver) to other tissues with a lower concentration (e.g., the cornea). In the current contribution, pathological tissue exhibited a strong positive correlation of NO and 3NT with GSSG%, the latter being the parameter that portrays the relation of GSSG (oxidized GSH) to total GSH. This indirectly demonstrates a plausible effect of S-nitrosylation on GSH metabolism and/or the release of GSH from tissues.

Hence, we have discovered that during the pathogenesis of pterygium, there is a protective response to the imbalance related to the decrease in production of NO by eNOS. This response consists of an increase in the level of GSH in the pathological tissue of pterygium through synthesis and/or mobilization. This capability of the organism to enhance the concentration of GSH in pathological tissue can be strengthened by presurgery systemic treatment with the cysteine precursor (NAC).

## 5. Conclusions

The protective response of the organism during the pathogenesis of primary pterygium is not clear. One of the possible sequences is that the pathological decrease in the activity of eNOS in pterygium tissue, and consequently in the bioaccessibility of NO and the S-nitrosylation of GSH or other nitrosothiols, results in a compensatory response to modulate the intracellular concentration of GSH by stimulating its synthesis and/or redistributing it between tissues.

## Figures and Tables

**Table 1:** Age (mean ± SD) of the study population by group and gender.

Groups	C	P	R	Ps	Pt
All patients	61.3 ± 6.0	56.9 ± 10.5	46.1 ± 6.3^*∗∗*^	53.0 ± 10.3	55.4 ± 9.3^*∗*^
N	19	36	19	17	29
Men	61.9 ± 5.9	53.8 ± 8.7^*∗*^	45.6 ± 7.1^*∗∗*&^	50.1 ± 8.0^*∗∗*^	56.5 ± 13.4^&^
N	7	18	10	8	11
Women	60.8 ± 5.4	62.1 ± 13.0	46.8 ± 4.9^*∗∗*^	55.5 ± 8.9	52.5 ± 10.3^*∗*&^
N	12	18	9	9	18

C, control group; P, untreated primary pterygium group; R, recurrent pterygium group; Ps, systemic-treated primary pterygium group; Pt, topic-treated primary pterygium group. Treatments were with NAC. ^*∗*^*p* < 0.05 versus the control; ^&^*p* < 0.05 comparing the primary pterygium group with other pathological groups.

**Table 2:** Bivariate Pearson correlation coefficients, starting from 0.8.

	All	Men	Women
GSH-CAT	0.949^*∗*^	0.962^*∗∗*^	0.940^*∗*^
GSH-GSSG	0.966^*∗∗*^	0.983^*∗∗*^	0.921^*∗*^
NO-GSSG%	0.992^*∗∗*^	0.905^*∗*^	
3NT-GSSG%	0.824		0.948^*∗*^
eNOS-NO	0.889^*∗*^		
eNOS-3NT	0.802		

^*∗*^ *p* < 0.05 and ^*∗∗*^*p* < 0.01, statistical significance of the correlation coefficients. Without ^*∗*^0.05 > *p* < 0.1 (tendency). Empty cells with *p* > 0.1.

## Data Availability

The data used and analyzed during the study are available from corresponding author on reasonable request.
